# Integrating enterprise risk management to address AI‐related risks in healthcare: Strategies for effective risk mitigation and implementation

**DOI:** 10.1002/jhrm.70000

**Published:** 2025-02-14

**Authors:** Gianmarco Di Palma, Roberto Scendoni, Vittoradolfo Tambone, Rossana Alloni, Francesco De Micco

**Affiliations:** ^1^ Department of Clinical Affair Fondazione Policlinico Universitario Campus Bio‐Medico Rome Italy; ^2^ Department of Law Institute of Legal Medicine University of Macerata Macerata Italy; ^3^ Italian Network for Safety in Healthcare (INSH) Coordination of Marche Region Macerata Italy; ^4^ Research Unit of Bioethics and Humanities, Department of Medicine and Surgery Università Campus Bio‐Medico di Roma Rome Italy

## Abstract

The incorporation of artificial intelligence (AI) in health care offers revolutionary enhancements in patient diagnostics, clinical processes, and overall access to services. Nevertheless, this technological transition brings forth various new, intricate risks that pose challenges to current safety and ethical norms. This research explores the ability of enterprise risk management as an all‐encompassing framework to tackle these arising risks, providing both a forward‐looking and responsive strategy designed for the health care industry. At the core of this method are instruments that together seek to proactively uncover and address AI‐related weaknesses like algorithmic bias, system failures, and data privacy issues. On the reactive side, it incorporates incident reporting systems and root cause analysis, tools that enable health care providers to quickly address unexpected events and consistently improve AI implementation procedures. However, some application difficulties still exist. The unclear, “black box” characteristics of numerous AI models hinder transparency and responsibility, prompting inquiries about the clarity of AI‐generated choices and their adherence to ethical benchmarks in patient treatment. The research highlights that with the progress of AI technologies, the enterprise risk management framework also needs to evolve, addressing these new complexities while promoting a culture focused on safety in health care settings.

## INTRODUCTION

### The rise of artificial intelligence (AI) in health care

AI represents one of the essential tools in the professional experience of numerous practitioners. Similarly, the health care sector has embraced this wave of innovation, adopting AI systems across various domains, from administrative management to advanced diagnostics. In this context, the European Union has opted to contribute scientifically by highlighting key risks to ensure the protection of patients and their families from potential harm arising from the use of intelligent systems in clinical practice.^1^ AI can bring numerous benefits to hospital settings, increasing diagnostic speed and accuracy. An algorithm can process vast amounts of data extremely quickly, facilitating the work of physicians and improving the quality of health care.^2^, ^3^, ^4^

### Challenges in managing AI‐related risks

Intelligent systems can support health care personnel in many ways, such as real‐time monitoring of vital signs with alert capabilities in case of changes, enabling more rapid interventions.^5^ Another application concerns the management of chronic diseases with algorithms that can suggest lifestyle modifications or propose adjustments to the therapeutic plan to better control the disease.^6^, ^7^ Furthermore, AI can facilitate access to health care by enabling health care professionals to reach patients in disadvantaged areas, indeed, services such as telemedicine allow for remote consultations and diagnoses.^8^, ^9^

### Challenges on the horizon

Despite the evident advantages, it is necessary to consider that the “indiscriminate” use of such systems, in the absence of controls, can create various issues, potentially shaping health care organizations negatively. For instance, intelligent systems can fall victim to so‐called “hallucinations,” that is, outputs containing totally or partially false information; thus, meticulous oversight of generated responses is required.^10^, ^11^ The introduction of such systems in health care demands an unconventional approach to risk management, and it is imperative for risk managers to develop new mitigation strategies to address the new risks associated with intelligent systems.^12^ As long as these algorithms operate in isolation, performing simple tasks independently, risks remain contained; issues are more likely to arise when intelligent systems must communicate with each other, managing complex tasks and exchanging data with a risk of error propagation.^13^

### Purpose and objectives of the study

In this scenario, enterprise risk management (ERM) presents itself as a promising solution to tackle the multiple and heterogeneous risks associated with the use of intelligent systems in health care settings. In 2004, the Committee of Sponsoring Organizations introduced the concept of ERM, presenting it as a viable alternative to optimize risk assessment and management using a holistic model as opposed to the traditional approach. The motto of this approach is “risk is capital,” underscoring how risks can generate profit opportunities rather than merely represent an economic threat. This approach was then adapted from the traditional corporate sector to health care, where the progressive corporatization of hospitals has made this model a valid alternative to the traditional clinical risk management approach.^14^ One of the fundamental concepts is that risk can be managed holistically and multidisciplinarily, avoiding management fragmentation that could lead to risk underestimation.^15^, ^16^, ^17^ The purpose of this article is to evaluate the risks associated with the introduction of intelligent systems in everyday clinical‐assistive practice, proposing an innovative management framework, such as ERM, to ensure appropriate mitigation of emerging risks.

## EMERGING RISKS OF AI IN HEALTH CARE SETTINGS

### Clinical risks

The risk of patient harm following the introduction of complex systems in health care represents a tangible threat; indeed, the integration of highly sophisticated technological tools within a high‐complexity setting, such as health care, can increase the likelihood of error. In the event of a malfunction in a traditional system, the damage can be contained; however, if highly integrated AI systems used for functions such as scheduling appointments, interpreting lab results or radiological images, and managing medical records experience failures, the consequences can be catastrophic.^18^ Despite technological advances in machine learning, clinical applications of AI systems still pose significant risks to patient safety, with multiple potential sources of error. First, predictive accuracy can be affected by “noise” in the input data; an error in the initial information is unlikely to be detected by the system, leading to error propagation. Even minor errors in the initial data can result in inaccurate clinical decisions. For instance, if the data distribution used as input in everyday clinical practice differs from that used to initially train the algorithm, erroneous outputs may be produced. This issue is particularly pronounced when AI systems are applied in settings different from those they were originally trained on.^19^ In fact, a study on pneumonia diagnosis found a decrease in AI performance when tested in a hospital setting different from the experimental one, while this suggests potential bias related to individual hospitals, the harm that can arise when evaluating different ethnicities is markedly higher.^20^ Moreover, unlike humans, AI cannot quickly adapt to unforeseen circumstances in clinical settings; for example, artifacts in a radiographic image can generate false positives if assessed by an intelligent system. This type of error highlights the need for such systems to undergo thorough training to be considered effective and safe, with diverse and inclusive datasets covering various target populations, as well as prolonged validation in real‐world settings. In any case, they should be regarded solely as support tools rather than being entrusted with decision‐making roles in place of physicians, preserving human oversight to minimize the possibility of error.^21^ It is also clear that, for the system to maintain its validity over time, it must undergo dynamic training that accounts for new clinical scenarios. Therefore, a balance must be maintained between continuous algorithm updates and human supervision to ensure the timely identification of emerging issues.^21^, ^22^ This could be the example of an AI algorithm that was implemented in a hospital to help diagnose pneumonia. The system, initially validated in controlled environments, demonstrated high accuracy during testing. However, once implemented in a real clinical setting, the algorithm began to produce numerous false negatives. The problem was traced to noisy input data, including radiographic images with artifacts, which the system struggled to interpret correctly. As a result, several patients with pneumonia were misdiagnosed or their conditions were not detected in a timely manner. This led to delays in initiating appropriate treatment, causing serious medical complications and increasing pressure on the clinical team. The hospital's reliance on the algorithm without further validation highlighted the limitations of AI systems when moving from the experimental to the practical stage.

### Technical risks

Another risk associated with the use of intelligent systems in health care lies in the limited transparency of such tools. For example, the underlying logic of deep neural networks is often difficult, if not impossible, to interpret for both physicians and technical staff. This phenomenon is known as the “black box,” where these technologies, lacking “transparency,” complicate the verification of the accuracy of diagnostic or therapeutic processes, reducing health care personnel's trust in AI systems. This issue is typical of deep learning models, which, while able to generate statistically correct algorithmic decisions, may lack a clear and accessible explanation for physicians, who, due to their role, must be able to justify and implement therapeutic choices. Additionally, in cases where an error occurs, the lack of transparency may hinder understanding of the cause, risking a repetition of the same failure. Therefore, this lack of transparency not only endangers patient health but also raises ethical concerns regarding accountability in the event of errors.^23^, ^24^

Another technical risk involves potential bias within the training data of the intelligent system. If the system is trained on limited or unrepresentative data, this may lead to errors in diagnosis and proposed treatments, resulting in discrimination based on gender, ethnicity, or age. Many studies have highlighted that AI can perpetuate inequalities if not adequately trained. For instance, if an algorithm is trained on a predominantly white population sample, it may be less accurate in diagnosing diseases in other ethnicities. Thus, the quality of the data used by AI is essential for ensuring accurate clinical decisions.^25^

At the same time, health care data may be incomplete, erroneous, or even manipulated, introducing vulnerabilities into a system. In this context, cyberattacks pose a serious threat, as the processing and continuous exchange of large volumes of data is central to the operation of intelligent systems. A cyberattack or security breach could compromise data integrity, with potentially disastrous effects on the patient care process.^26^

The increasing use of AI in hospitals can lead to cognitive overload for physicians, who are required to manage an ever‐growing amount of information generated by intelligent systems. While automation in generating clinical responses may be helpful, it could reduce the physician's ability to critically exercise professional judgment. This risk is particularly evident in emergency settings, where health care personnel must make rapid and complex decisions. If the automated system fails, physicians may not be ready to intervene with alternative solutions, thereby increasing the risk of errors.

Another area of concern is the long‐term reliability of AI systems. Many technologies require continuous updates and maintenance to remain accurate. The lack of system updates or hardware obsolescence can reduce effectiveness over time.^20^ Thus, an algorithm that is not retrained with new data could become obsolete, resulting in incorrect diagnoses. Additionally, maintaining intelligent systems requires resources and technical expertise that may not always be available in hospital settings, adding a further layer of complexity.^2^, ^21^, ^27^ An illustrative example is that of an AI‐based telemedicine platform implemented to improve patient monitoring and remote consultations. At an early stage, the system functioned suitably, demonstrating efficiency in processing and managing large volumes of data regarding patients. Subsequently, it was the subject of a cybersecurity breach, during which unauthorized individuals gained access to sensitive patient data. The cyber‐attack exploited vulnerabilities in the system's data encryption protocols, resulting in, among other things, the disruption of clinical operations, with medical staff forced to revert to manual processes, significantly slowing patient care and increasing operational strain. This resulted in reputational damage to the company as well as legal repercussions for any damages achieved by patients.

### Socio‐ethical risks

As previously discussed, AI systems in health care rely on large datasets that may reflect societal biases. Historically marginalized groups, such as ethnic and racial minorities, may be underrepresented or misrepresented in these datasets, resulting in suboptimal care. Unbalanced datasets may lead to inaccurate predictions for these groups, exacerbating existing health disparities. Gender biases are also evident: reports of pain from female patients are often downplayed, and these biases could be perpetuated or amplified by AI. Another crucial issue is that of “health care deserts,” or areas where vulnerable populations lack access to health care services. This issue could be worsened by the digital divide, as populations without access to technology or digital literacy may be excluded from clinical advances such as telemedicine. Thus, marginalized communities may lack the infrastructure or resources needed to benefit from AI technologies, turning these advancements into barriers for certain populations.^28^, ^29^, ^30^ Another key element for ensuring AI systems are fair is to build social trust. Without transparency, explainability, and ethical marketing, people may distrust these technologies. It is essential to design AI systems with user‐centered values and to communicate their capabilities clearly to the public to encourage acceptance. Addressing AI biases requires interdisciplinary collaboration, continuous evaluation, and transparency. Diverse teams of AI developers, clinicians, ethicists, and even patient advocacy groups can help ensure that AI tools are designed inclusively and equitably. Proper representation in datasets, careful data labeling, and monitoring AI systems in real health care settings is essential to prevent the perpetuation of biases.^21^, ^31^, ^32^, ^33^ One notable example might involve an AI system designed to prioritize emergency patients based on the severity of their conditions. Although the system performed well in controlled test environments, its implementation in a real‐world hospital setting revealed consistent problems; in fact, the algorithm underestimated the severity of conditions of patients of some specific ethnicities due to the lack of heterogeneity in the training dataset. These biases stemmed from the lack of representation of different populations, leading to delays in care for some patients and exacerbating existing health disparities. This failure not only put affected patients at risk but also raised ethical concerns about fairness and equity in AI‐driven health care decisions. The incident highlighted the crucial need for diverse and representative datasets to ensure equitable care outcomes for all demographic groups.

### Privacy and data protection

The increasing use of AI in health care, accelerated during the SARS‐CoV‐2 pandemic, has raised concerns about data privacy, confidentiality, and the protection of patients’ rights. These risks include the exposure and potential misuse of sensitive data, which could lead to violations of individual rights as well as nonmedical uses of personal information. Informed consent remains a critical issue in this context, as patients must be adequately informed to make informed decisions about sharing their health data. Specifically, the introduction of nontransparent AI algorithms, along with complex consent forms, may limit patient autonomy, creating an obstacle to the shared decision‐making process with physicians. Such complex algorithms not only make it difficult for patients to understand data‐sharing practices but also create new vulnerabilities in terms of cybersecurity. The risk of cyberattacks, as well as unauthorized data usage, raises particular concerns, with issues including privacy breaches, identity theft, and the potential targeting of AI‐controlled medical devices. Some studies highlight the need for ethical and transparent integration of AI technologies in compliance with GDPR to ensure that data are used securely and in accordance with regulations, thereby reducing the risk of system failures or algorithmic biases.^34^ Another significant risk relates to privacy violations involving the use of big data. Many health care facilities have already encountered issues related to data protection during cyberattacks; some have shared or stored sensitive health data on publicly accessible servers, resulting in unauthorized exposure of information^35^ Although data used for research purposes should be anonymized, elements such as treatment dates, medical notes, or personal information have sometimes not been removed. This type of exposure poses a significant risk, particularly with the use of AI, which requires large amounts of data to function effectively. Unauthorized access to health data can also facilitate insurance fraud, highlighting the vulnerability of these infrastructures.^36^ Obtaining informed consent for the use of patient data is a topic of intense ethical debate. Generally, patients provide consent for data usage when admitted for medical treatment or billing purposes. However, when data are used for research or purposes unrelated to therapy, separate consent is required. If data have been anonymized, GDPR no longer applies, allowing health care facilities to use these data without additional legal restrictions.^37^ However, even though anonymized data should theoretically be protected, there are concerns that combining these data with other sources could make it possible to identify individuals.^38^

Proposed solutions include enhancing the security of cloud storage systems and raising awareness among health care providers about the risks associated with these technologies to create a health care environment compatible with the safe and responsible use of AI. The growing complexity requires collaboration among researchers, policymakers, and health care professionals to develop more robust regulations and more effective security mechanisms to prevent privacy breaches and ensure the ethical and secure use of AI in health care.^39^, ^40^ Nonetheless, if data have been anonymized, GDPR no longer applies, allowing health care facilities to use such data without further legal restrictions.^37^ Still, there are concerns that combining anonymized data with other sources might enable the re‐identification of individuals.^38^ One possible example involves the implementation of an AI system to monitor COVID‐19 patients, optimizing care delivery. Although the system improved patient management, a critical problem emerged when sensitive health data was mistakenly shared with an unauthorized third party. The breach occurred due to poor data access controls and led to the exposure of the personal medical information of thousands of patients; this not only resulted in fines for noncompliance with data protection regulations but also undermined patients' confidence in the hospital's ability to safeguard their information. The situation has highlighted the urgent need for robust data governance, including stricter access protocols and increased staff training, to ensure compliance with privacy standards and protect sensitive health data.

## DISCUSSION

### Enterprise risk management: A holistic approach to AI challenges

**FIGURE 1:**
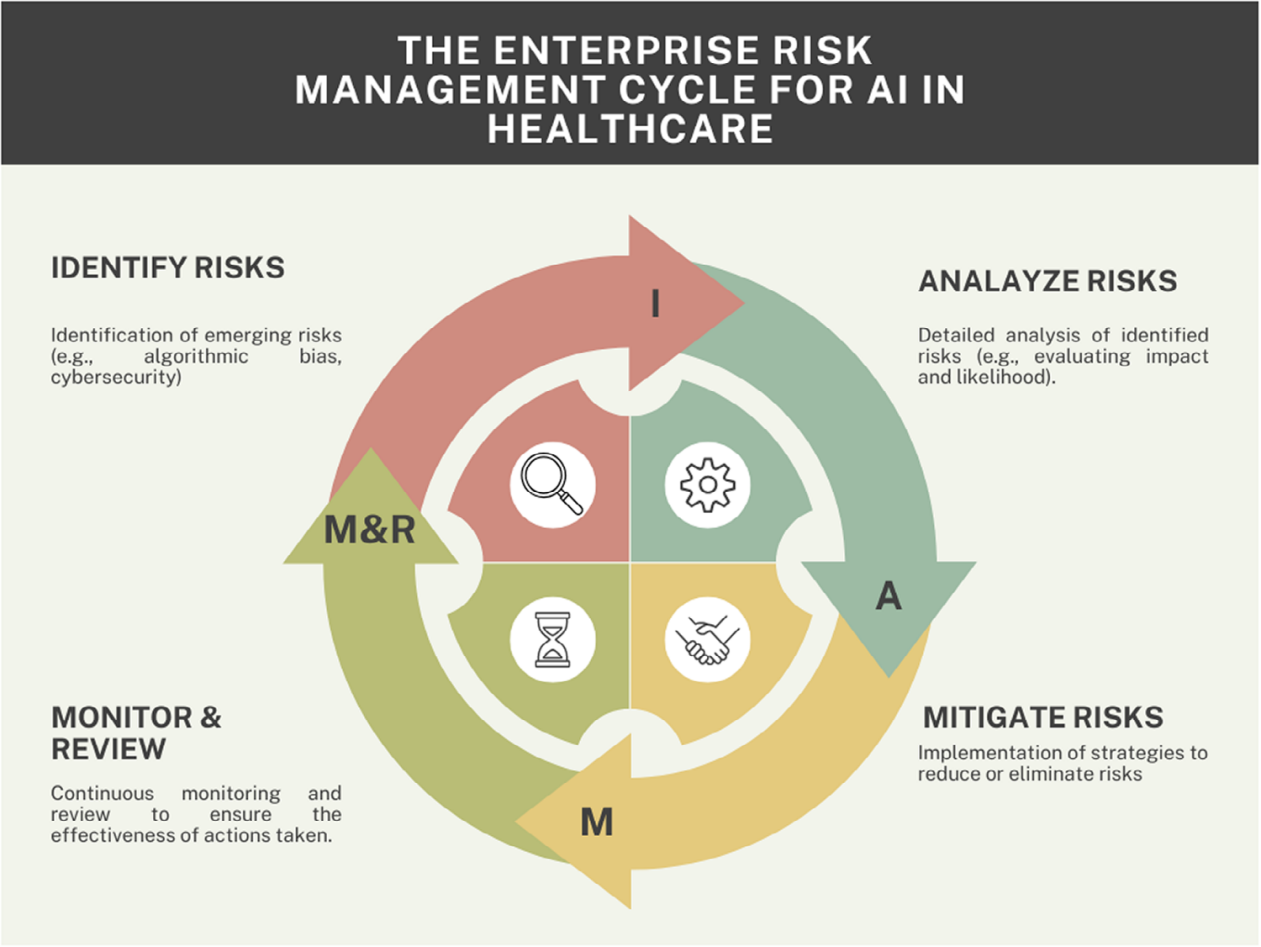
Illustrates the stages of ERM—identification, analysis, mitigation, and monitoring—applied to AI‐related risks in health care.

To integrate enterprise risk management (ERM) into managing the risks associated with using intelligent systems in health care, the key principles of ERM can be utilized to address emerging challenges related to AI technologies.^41^ ERM provides a holistic approach to identifying AI‐related risks, including those associated with system malfunctions, privacy breaches, and algorithmic biases, which can compromise patient safety and data integrity (Figure 1). ERM's management approach involves shared tools like brainstorming, focus groups, and questionnaires to gather insights from various operational units involved. This approach can help identify potential risks across different areas, from clinical to technological and financial risks, allowing for a detailed risk analysis.

### Preventing and correcting AI‐related errors: An ERM perspective

Proactive methods such as FMECA (Failure Mode, Effects, and Criticality Analysis) are essential for anticipating and mitigating potential AI system failures, ensuring the safe and responsible use of technology. This tool operates on the premise that some errors are inevitable and may occur with certain probabilities; through in‐depth analysis, the primary goal is to identify all potential ways an AI system failure, error, or malfunction might occur. This enables prioritizing critical situations and optimizing the system or process to improve safety and reduce failure risks.^42^ Applying FMECA minimizes defects or malfunctions that may arise from inadequate design or risk oversight during the initial phase, thus strengthening the safety and reliability of the intelligent system by anticipating operational issues and enhancing overall performance. Simultaneously, reactive methods like incident reporting and peer review help identify and mitigate risks once they materialize, enabling a swift response and a process analysis to prevent recurrence. Other reactive methods include root cause analysis (RCA), which identifies the root causes of potential AI system malfunctions, and Hazard and Operability Study (HAZOP), which can be applied to analyze complex scenarios, ensuring that each potential risk is identified and mitigated before negatively impacting clinical safety.

### Risk mapping in AI‐enhanced health care systems

**FIGURE 2:**
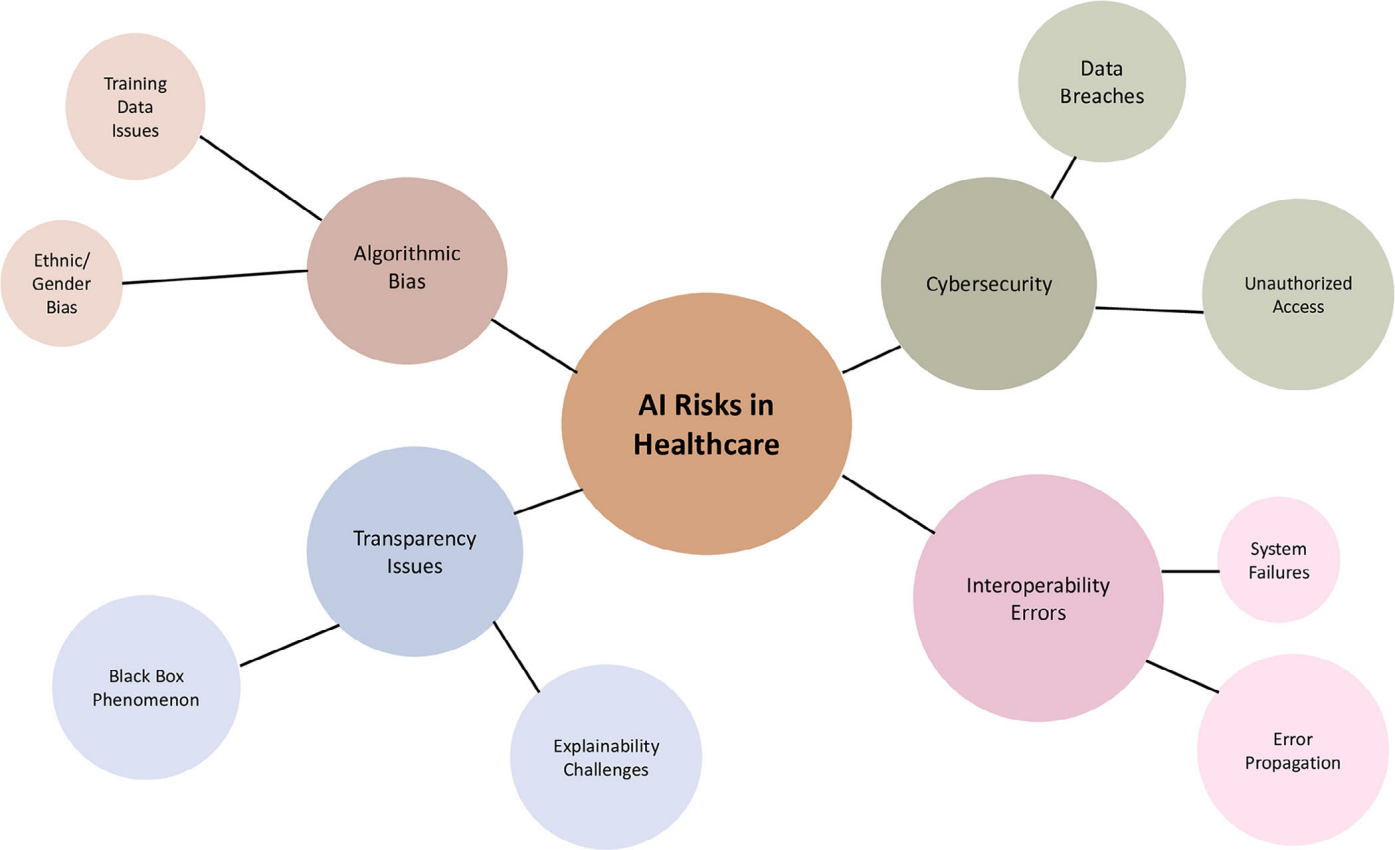
Depicts key AI‐related risks in health care, including algorithmic bias, cybersecurity, and transparency issues, with their subcategories.

Such tools are crucial for managing the risks associated with AI implementation. Once AI‐related risks are identified, ERM techniques should be applied for risk mapping, especially as less transparent technologies, like deep neural networks, pose serious clinical and decision‐making liability risks.^43^ (Figure 2). Mapping these risks and prioritizing them based on a scale of probability and impact is crucial for developing appropriate mitigation strategies. For example, cybersecurity risks may receive a high priority score due to potential legal and reputational implications. ERM offers various risk control techniques to address AI‐related risks, such as risk control (limiting AI use in clinically sensitive areas), risk reduction (introducing backup systems and manual checks), and risk transfer (insurance against AI‐related errors or damage from cyberattacks). This approach is essential to reduce the negative impact of potential AI malfunctions, preventing catastrophic errors, and protecting patients’ sensitive data.^44^

### Driving safety through monitoring and collaboration

Finally, the ERM process concludes with implementing corrective measures; however, it is necessary to constantly monitor key risk indicators (KRIs) to detect any changes in the probability and impact of AI‐related risks promptly. KRIs are metrics used to monitor and measure an organization's risk level over time, providing early warning signals of potential risk events that could negatively impact operations or objectives.^45^ KRIs are fundamental to risk management, allowing the risk manager to identify and address potential issues before they occur. Additionally, KRIs differ from Key Performance Indicators (KPIs) as KRIs focus on risk management, whereas KPIs monitor performance. In essence, KRIs measure factors that may negatively impact goal achievement and are often linked to specific risks. For instance, monitoring AI algorithm performance over time is essential, ensuring that systems are updated and operate on datasets representative of the target population to avoid biases or clinical errors.^46^ Mitigating secondary risks associated with intelligent systems requires collaboration among various professionals, including Data Protection Officers (DPOs) for GDPR compliance and data breach risk management, AI experts and computer engineers for technical maintenance and model training, and ethicists to address the moral implications of using AI in health care. In this multidisciplinary context, the Risk Manager plays a crucial role as a facilitator and a link between different disciplines, coordinating risk management processes, fostering cooperation among working groups, and promoting a safety‐oriented organizational culture that supports innovation.^47^

### Future directions for ERM in AI risk management

However, despite ERM providing numerous tools and resources for a thorough management of risks associated with intelligent systems in health care, several open issues remain that this management approach will need to address in its initial application stages. Specifically, ERM should pay particular attention to the interpretability of AI models; while deep neural networks offer high performance, their inherent complexity makes it difficult to understand the rationale behind decisions. Therefore, it is crucial to develop techniques to make models more transparent and interpretable, facilitating risk assessment and increasing end‐user trust.^48^ ERM must also proactively address algorithmic bias to prevent discrimination and unfair decisions, implementing mechanisms to identify and mitigate biases in training datasets and within the models themselves, ensuring fairness and inclusivity. Additionally, the use of AI in health care raises important issues regarding legal liability. ERM must help define accountability in cases of errors or harm caused by AI systems, highlighting the need for clear legal and ethical frameworks to ensure transparency and accountability.^49^ Indeed, such a risk management model should promote a safety culture within the organization, engaging all employees in training and awareness activities about AI‐related risks. ERM is not a static process but a dynamic one, necessitating continuous evaluation of the effectiveness of risk control measures and updating the risk management plan based on new information and technological advancements. Finally, ERM itself can benefit from using advanced tools like big data analysis and machine learning to identify emerging risks and improve adverse event prediction.^50^, ^51^

## CONCLUSIONS

The use of ERM within health care organizations that have chosen to implement AI in their clinical assistance pathways could be an effective solution for better managing the risks associated with the use of intelligent systems in daily practice. ERM's holistic approach enables the identification, evaluation, and mitigation of all risks arising from the use of AI systems, including technical malfunctions, patient privacy breaches, and algorithmic biases. In this context, the use of both proactive risk assessment tools, such as FMECA, which anticipate potential problems, and reactive tools like RCA and HAZOP, which correct errors within internal processes, will be increasingly effective in optimizing clinical‐assistance processes in response to critical events. Continuous monitoring of KRIs will be essential in this context, allowing timely detection of changes in the likelihood and impact of risk events. This approach provides an ongoing assurance of safety, aligning with the highest clinical standards in real‐time.^52^, ^53^, ^54^

However, despite the clear benefits, many significant challenges lie ahead. For instance, increasingly complex neural networks will make result interpretation more difficult, with less transparent clinical decision‐making pathways due to the “black box” phenomenon. This system “opacity” could lead to increased medical‐legal disputes, necessitating the development of algorithms that operate more “transparently.” Another crucial issue to address is algorithmic bias, as this could paradoxically lead to increased health care disparities.^55^, ^56^, ^57^ This suggests that in the future, the risk management framework will need to embrace technological advancements, incorporating intelligent systems capable of providing more accurate risk analyses.^58^ Lastly, this impending change will require regulatory review to define liability limits in case of errors.

Thus, while ERM represents an essential and comprehensive approach to managing risks associated with AI implementation in health care, much remains to be done to ensure the safe integration of intelligent systems in hospital settings.

## CONFLICT OF INTEREST STATEMENT

The authors declare that they have no conflicts of interest.
